# Pan-cancer analysis of neoepitopes

**DOI:** 10.1038/s41598-018-30724-y

**Published:** 2018-08-24

**Authors:** Gabriel N. Teku, Mauno Vihinen

**Affiliations:** Department of Experimental Medical Science, BMC B13, Lund University, SE-22184 Lund, Sweden

## Abstract

Somatic variations are frequent and important drivers in cancers. Amino acid substitutions can yield neoantigens that are detected by the immune system. Neoantigens can lead to immune response and tumor rejection. Although neoantigen load and occurrence have been widely studied, a detailed pan-cancer analysis of the occurrence and characterization of neoepitopes is missing. We investigated the proteome-wide amino acid substitutions in 8-, 9-, 10-, and 11-mer peptides in 30 cancer types with the NetMHC 4.0 software. 11,316,078 (0.24%) of the predicted 8-, 9-, 10-, and 11-mer peptides were highly likely neoepitope candidates and were derived from 95.44% of human proteins. Binding affinity to MHC molecules is just one of the many epitope features. The most likely epitopes are those which are detected by several MHCs and of several peptide lengths. 9-mer peptides are the most common among the high binding neoantigens. 0.17% of all variants yield more than 100 neoepitopes and are considered as the best candidates for any application. Amino acid distributions indicate that variants at all positions in neoepitopes of any length are, on average, more hydrophobic than the wild-type residues. We characterized properties of neoepitopes in 30 cancer types and estimated the likely numbers of tumor-derived epitopes that could induce an immune response. We found that amino acid distributions, at all positions in neoepitopes of all lengths, contain more hydrophobic residues than the wild-type sequences implying that the hydropathy nature of neoepitopes is an important property. The neoepitope characteristics can be employed for various applications including targeted cancer vaccine development for precision medicine.

## Introduction

The task of the immune system is to detect and destroy foreign molecules and organisms. This is achieved by the numerous mechanisms and processes that form the innate and adaptive arms of the immune system. Three complementary adaptive systems have evolved to recognize foreign materials. First, antibodies recognize and neutralize non-self-molecules. Second, the major histocompatibility complexes (MHCs) I and II bind to and present short fragments of foreign peptides to T cells. Third, T cell receptors are produced with a similar recombination process as antibodies. The binding sites of these molecules are highly variable due to genetic recombination processes. Therefore, it is essential that the immune system does not react against natural human molecules to prevent autoimmune diseases. Safeguards against self-reactivity and induced tolerance prevent this from happening. These mechanisms are still poorly understood. Recently, antigen-specific regulatory T-cells were shown to be responsible for autoimmunity protection^1^.

Variations accumulate during a lifetime. It has been estimated that in fibroblasts, B, and T cells, the mutation rate is 2–10 variations per diploid genome per cell division^2^. This means that normal cells can have from hundreds to several thousands of variations in comparison to the original genome of the individual^3^. In cancers, the variation rate can be much higher, for example, lung cancer cells typically contain over a million variants^4^. It is thus highly likely that cancer tissues include numerous immunogenic proteins because substitutions in the DNA, the most abundant changes in cancers, can lead to amino acid substitutions (AASs) in proteins. Such immunogenic epitopes are called neoantigens.

To use neoantigens for therapeutic purposes, numerous research projects aim at detecting cancer variant peptides for diagnosis and treatment, including vaccination. Although next-generation sequencing methods are efficient for sequencing and detecting variants in tumors, the translation to neoantigens is not straightforward. Neoantigen-based treatment would facilitate personalized medicine for cancer patients. In addition to the possibilities for treatment, neoantigens could possibly be used for diagnosis especially in the case of relapse.

Numerous methods have been developed to predict the antigenicity of peptides, especially those binding to MHC type I molecules^5^. The performance of these tools varies^6,7^ depending on the size and composition of the used benchmark dataset^8^. Despite intensive research, the number of experimentally defined epitopes is still relatively small^7,9^ and affects the performance of the predictors. By combining the epitope predictions with experimental validation assays, the performance can be improved. NetMHC^10,11^ is a predictor for epitopes and available in several versions for different purposes. It has consistently been among the best tools in performance assessments^7^.

Neoantigen load and occurrence in several cancers has been widely studied^6,12–16^, however, the detailed pan-cancer analysis of the occurrence and characteristics of neoantigens has been missing. We investigated proteome-wide amino acid substitutions in 30 cancer types. First, we predicted the most highly likely neoepitope candidates. This was done by comparing the binding affinities of the wild-type and variant-containing peptides. The peptides with high binding neoepitopes were investigated at many levels including HLA distributions, Gene Ontology distributions of the proteins and functions of the proteins with the largest numbers of neoepitopes, distribution in cancer types, as well as distributions at amino acid level. 11,316,078 (0.24%) of the predicted 8-, 9-, 10-, and 11-mer peptides were highly likely neoepitope candidates and originated from 95.44% of human proteins. A very small ratio (0.17%) of variants was predicted to lead to the formation of more than 100 neoepitopes. Amino acid distributions, at all positions in neoepitopes of all lengths, contain more hydrophobic residues than the wild-type sequences. The ubiquitous neoepitopes and the hydropathy nature of neoepitopes can be taken advantage of in cancer vaccine development. This represents the first large scale neoantigen distribution study that sheds light on the nature of neoepitopes across cancer types.

## Results

We performed extensive pan-cancer analysis of neoepitopes and estimated the number of variants that lead to an immune response. The dataset for 30 cancer types^4^ contained in total 783,615 AASs. For each AAS, a 21-mer parent peptide was constructed such that the variant position was at the center of the peptide, flanked by ten amino acids on both sides. In human, MHC molecules are also called human leukocyte antigens (HLAs). We predicted with the NetMHC 4.0 software^10^ binding affinity of peptides of lengths 8, 9, 10 and 11 residues for 80 HLAs. The reason for focusing on the MHC I system was that MHC II predictions are less reliable because the motifs are more promiscuous, longer and more variable^17^. The variants were tested in all the sequence positions for each peptide length. These accounted for a maximum of 38 variant peptides per parent peptide. Similarly, for comparison of binding affinities, we obtained predictions for the corresponding wild-type peptides. Altogether, we performed 4,706,079,200 predictions (Table 1).

**Table 1:** Summary of predictions made with NetMHC 4.0.

Feature	Value
Number of variants	783,615
Number of predictions	4,706,079,200
Number of proteins with variants (% proteome)	18,324 (89.55%)
Number of weak binding peptides (%)	66,015,404 (1.40)
Number of high binding peptides (%)	21,712,146 (0.46)
Number of predicted neoepitopes (%)	11,316,078 (0.24%)
Number of neoepitopes per protein (max; mean; min)	42,930; 618; 1
Number of neoepitopes per cancer (max; mean; min)	1,972,000; 377,200; 859
Number of neoepitopes per variant (max; mean; min)	231, 15, 1
Number of proteins with neoepitopes (% all proteins)	18,311 (89.15%)
Number of variants that cause neoepitopes (%)	747,856 (95.44)

### General properties of predicted peptides

Computational studies of neoantigens are based on predicted affinity to MHC molecules. Similar to previous studies IC_50_ value of <500 nM was used to indicate weak binding and <50 nM high binding peptides^18^. From the predictions, we selected high and weak binders and investigated them further.

**Figure 1:**
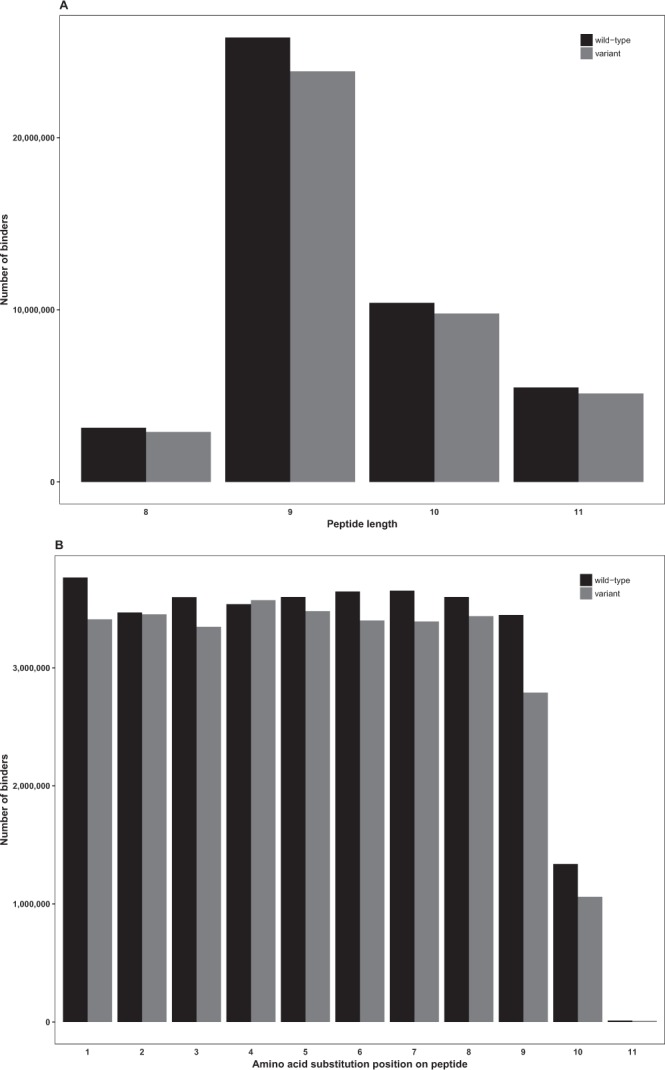
Distribution of 8- to 11-mer binding peptides and AAS distribution at positions 1–11. (**A**) Distribution of predicted peptide binders of lengths 8, 9, 10, and 11. (**B**) Distribution of the position of AASs in the peptide binders. The low number of AASs at position 11 is due to the very small number for 11-mer peptides because AASs at position 11 can only occur in 11-mers. In both panels, wild-type peptides are indicated in black and variant peptides in grey.

The numbers of wild-type (41,667,139) and variant (44,853,374) binders (both weak and high binding) were quite similar. Thus, many natural human sequences have high affinity at least to one common HLA molecule. Our analysis concentrated on MHC type I peptides, which have a strong preference for short 8 to 11 residue-long peptides. There were more 9-mer peptides among the binders than peptides of the other lengths combined. The 9-mers were by far the most abundant predicted binders (>57%) followed by 10-mers (Fig. 1A). The 8-mer peptides were the least frequent (6.9%). The distributions in the n-mers were similar for the peptides with both wild-type and variant sequences.

**Figure 2:**
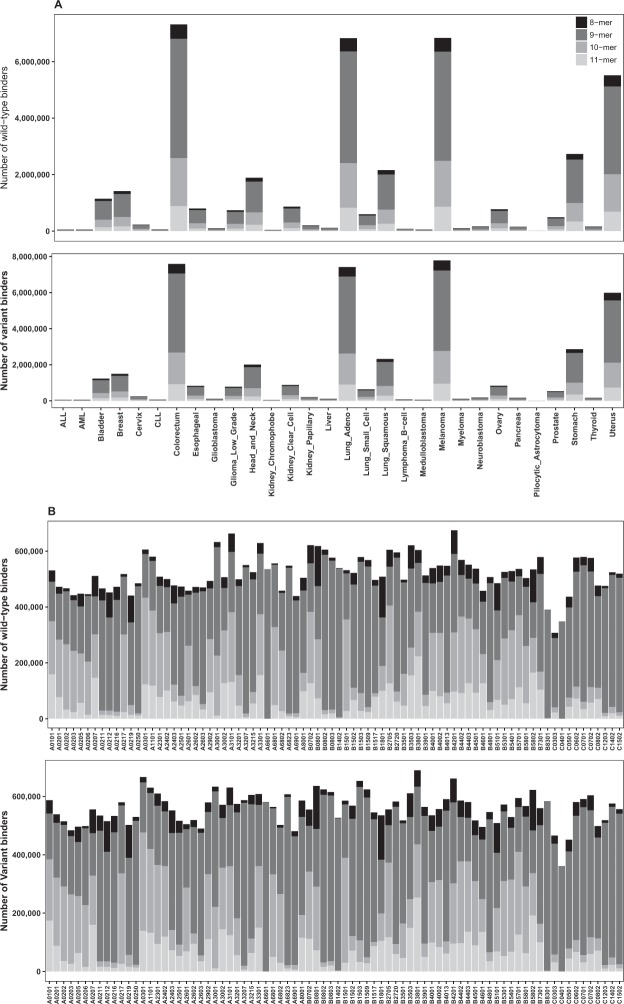
The distribution of peptide binders across cancer types and HLAs. (**A**) Distribution of predicted peptide binders across cancer types. The numbers of binding peptides are very similar for the wild-type and the variant datasets. The proportion of binders follows the overall rate of variations in the cancer types. (**B**) Distribution of predicted peptide binders across HLAs. The binders were distributed evenly across the HLAs.

Together the weak and strong binders represented 1.8% of all the possible and predicted peptides (Table 1). The distribution of AASs at peptide positions 1 through 11 were very similar across the wild-type and variant datasets (Fig. 1B). The number of binders with a variant at position 11 was diminutive compared to the other positions, in both the dataset for wild-type and for variant peptides. This was due to a low number of binders of length 11, and to the fact that this position appeared only in the longest peptides. There were no differences in the distributions of binders among the cancer types for both the wild-type and variant peptides (Fig. 2A). Colorectum, lung adenocarcinoma, melanoma and uterine cancers contained the largest numbers of binders, whereas there were only a few binders in acute lymphocytic leukemia (ALL), acute myeloid leukemia (AML), chronic lymphocytic leukemia (CLL), kidney chromophobe and pilocytic astrocytoma. These trends closely followed the overall rate of variations in the cancers. Next, we investigated the frequencies of the peptide binders to HLAs (Fig. 2B). The binders were distributed almost evenly across the HLAs. However, numbers of binders for HLAs B8301, C0303 and C0401 were clearly smaller than for all the others, in both datasets.

In conclusion, these results indicate that the 9-mers were the most common binding peptides and there were practically no major differences in the distributions for wild-type and variant peptides. Thus, numerous normal human peptides were predicted to be antigenic, which indicates a limitation for the used prediction method. However, the binding affinity is not the only factor that contributes to the T cell response. As discussed below, we aimed at solving the overprediction problem in further studies.

### Properties of high and weak binding peptides

Both weak and high binding peptides with AASs have been considered as neoepitopes in some previous studies^19,20^. The numbers of high binding wild-type and variant peptides were 10,314,928 (0.22% of all peptides) and 11,189,860 (0.24%), respectively, and those for weak binders were 31,352,211 (0.67%) and 33,663,514 (0.72%), respectively. The distribution of AASs to positions 1–11 on both the wild-type and variant peptides and in both the high and weak binders was quite similar (Fig. S1). The distributions to amino acid positions were very even throughout the datasets. The only exceptions were positions 10 and 11, as expected since these positions could only occur in 5% and 2% of all possible positions, respectively.

The proportions of wild-type and variant binders across the cancer types (Fig. S2) were similar to those for all binders (Fig. 2A). The distribution of weak and strong binders within HLAs in both the wild-type and variant datasets (Fig. S3) followed very closely that for all binders (Fig. 2B). The 9-mer peptide binders were clearly the most abundant followed by 10-, 11- and 8-mers, in that order, across all the HLAs. There were differences between HLAs in the ratios as well as the amounts of predicted peptides. The HLAs B0801, B1801 and B5802 have relatively many predicted 8-mer binders. For some HLAs, almost all the peptide binders were 9-mers, such as A6601, B1402, and C0401. Further, HLAs A0217, A0301 and B1501 had many 10-mers, while A0101 and B3801 had many 11-mer binders.

These data show that the high and weak binding peptides had very similar characteristics, and this applies equally to the wild-type and variant-containing peptides.

### Properties of neoepitopes

We defined neoepitopes as variant peptides with a high binding affinity (≤50 nM) to an HLA and for which the corresponding wild type sequence has either weak affinity (>50 and ≤500 nM) or is not predicted to bind at all. This is a stricter requirement than used in some earlier studies^12,15,21^. The results for the wild-type and variant binders indicated that there were no qualitative differences between the groups. Here, we concentrated on the most likely neoepitopes and therefore restricted the further studies to neoepitopes defined this way. From both the variant and wild-type datasets, 66,015,414 peptides had a weak affinity, and 21,712,146 had a high affinity. Altogether, there were 11,316,078 (0.24%) neoepitopes, which covered 95.44% of the tested variants and 89.15% of the encoded proteins. The percentage of predicted neoepitopes was rather small, but since the number of tested peptides was enormous, about 2.4 billion variant peptides, there were still a substantial number of peptides left. According to these results, practically every human protein-coding gene would code for neoantigens. This is likely not true, as discussed below.

**Figure 3:**
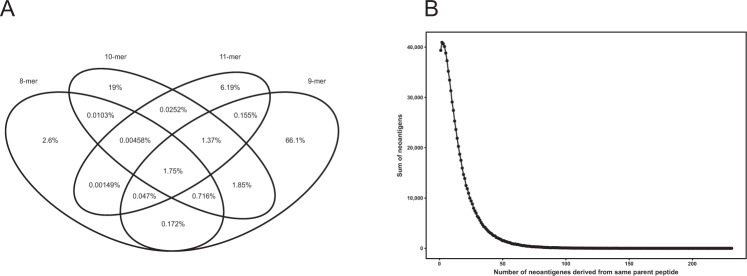
The overlap between 8-mers to 11-mers and distribution of neoepitopes per variant. Venn diagram of the overlap between the predicted peptide binders of lengths 8–11. 9-mers were the most common among neoepitopes. (**A**) Variant that gave rise to a neoepitope of a certain length very seldom formed neoepitope even when just one residue is added or deleted from the sequence. (**B**) The distribution of neoepitopes per variant peptide. The number of neoepitopes per variant varied widely, from 1 to 231. Most variants yielded just one or a few neoepitopes. Only a small fraction of variants appeared in many epitopes.

Altogether 72.26% of the neoepitopes were 9-mers, 22.06% 10-mers, 8.52% 11-mers and 4.73% were 8-mers. Only 1.75% of the variants gave rise to neoepitopes of all lengths (Fig. 3A). In total, only 6.1% of the variants gave rise to neoepitopes of at least two peptide lengths. This is a rather small number and indicates that the requirements for different peptide lengths vary. Thus, a variant that gave rise to a neoepitope of a certain length very seldom formed neoepitope of another length even when just one residue is added or deleted from the sequence. This result also indicates that NetMHC predictions were specific for peptide length. Predictions of just 9-mers would yield 72.26% of all neoepitopes and save a substantial amount of time when investigating large datasets as in here.

The number of neoepitopes per variant varied widely, from 1 to 231 (Fig. 3B). Most variants yielded just one or a few neoepitopes. Only a small fraction of variants appeared in many epitopes. 1282 variants (0.17%) among the neoepitopes occurred ≥100 times (Table S1). These could be called super-epitopes. The proteins that yielded the largest number of neoepitopes were listed in (Table S2). These included TP53, PTEN, as well as many olfactory receptors and follicular dendritic cell secreted protein (FDCSP).

**Figure 4:**
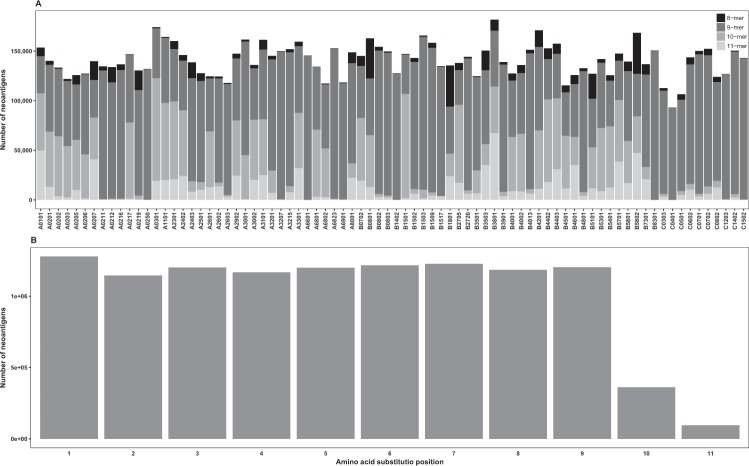
The distribution of neoepitopes among HLAs and to AAS at positions 1–11 in neoepitopes. (**A**) The distribution of neoepitopes among HLAs. The differences in the overall numbers of neoepitopes per HLA were minor. 9-mers are most common. (**B**) The distribution of AASs at positions 1–11 of the neoepitopes. The split down to the positions of variants within the peptides indicated very even distribution except for the small ratios for positions 10 and 11.

There were minor differences in the overall numbers of neoepitopes per HLAs (Fig. 4A). The results indicate that 9-mers were the most common among neoepitopes (compare with Fig. 3A). 10-mers had significant shares, e.g. in A0217, AO310, and B1501. The largest share of 11-mers appeared in B3801 and C1502. Neoepitopes for A0250, A3207, A6601, B1402, B8301, C0401, C1203, and C1502 consisted almost entirely of 9-mers, while in B1801 the largest number is for 8-mers.

The distribution of AAS at positions 1–11 in the neoepitopes (Fig. 4B) was similar to that of all binders, as well as high and weak binders (Fig. S1). The split down to the positions of variants within the peptides indicated very even distribution except for the small ratios for positions 10 and 11.

**Figure 5:**
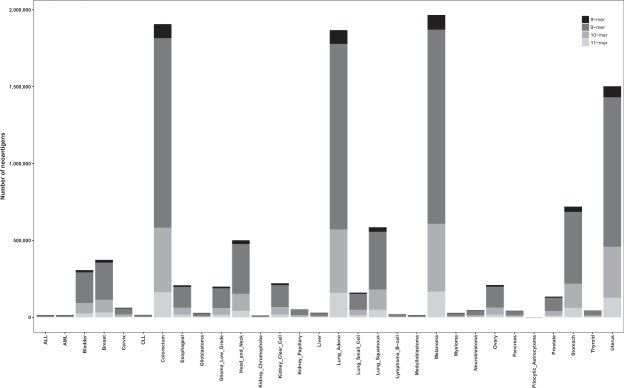
The distribution of neoepitopes across cancer types. Melanoma, colorectum cancer and lung adenocarcinoma have the largest numbers of neoepitopes, which correlates with the number of ASSs that occur in these cancers. Although the cancer variants originate due to different mutation mechanisms, depending on the type of cancer, the distributions of neoepitopes and peptide lengths were similar.

The distribution of predicted neoepitopes across cancer types is shown in Fig. 5. Melanoma, colorectum cancer, and lung adenocarcinoma had the largest number of neoepitopes, 17.32%, 16.88% and 16.65% of all neoepitopes, respectively. This was expected as melanoma, colorectum cancer and lung adenocarcinoma represent 17.12%, 16.94% and 16.33% of all the AASs, respectively. The distributions of peptide lengths were consistent throughout the cancer types. Although the cancer variants originate due to different mutation mechanisms, depending on the type of cancer, the distributions of neoepitopes and peptide lengths were similar.

The HLA-specific neoepitope distributions for each cancer type are shown in Fig. S4. The distributions across the cancer types were overall very similar. This visualization indicates differences in HLAs irrespective of the total numbers of variants which varied very widely. In the cancers with many cases, the HLA patterns are very similar (e.g., melanoma, colorectum cancer, and lung adenocarcinoma). In the case of cancers with low numbers of cases and variants, we could see discrepancies for some HLAs compared to general patterns. See for example kidney chromophobe cancer, pilocytic astrocytoma, and B-cell lymphoma. The reason for pilocytic astrocytoma differing from the others was that there are just a small number of AASs (178) and consequently only a few neoepitopes.

To study the distribution of neoepitopes in the cancer patients from which the AAS data used in this study was derived, we mapped the neoepitopes to the patient data. Fig. S5 indicates that there were huge differences in neoepitope numbers among patients in the cancer types. The minimum and maximum numbers of neoepitopes per patient were 4 and 529,280, respectively. The mean and median numbers of neoepitopes per patient were 6,856 and 1,992, respectively. The cancers with the largest number of neoepitopes included colorectum, lung adenocarcinoma, melanoma, stomach, and uterus cancers. These are the tumors with the highest mutation burden, as expected.

Table S1 contains the list of variants with the largest number of neoepitopes sorted in descending order. The top of the list includes follicular dendritic cell secreted protein (FDCSP), cytochrome c oxidase subunit VIIc (COX7C), transmembrane superfamily members (TM6SF1 and TM9SF4), dehydrogenases (SDR16C5, HSD3B1, ACAD10), receptors (VN1R2, P2RY1, TACR3, GPR141) and phosphatases (PPP3CA, PTEN, PPAP2A) linked to carcinogenesis.

The most frequent variants originate from proteins that have catalytic activity (30.2%), transporter activity (22.9%) or are involved in binding (19.1%). These proteins include follicular dendritic cell secreted protein (FDCSP), cytochrome c oxidase subunit VIIc (COX7C), phosphatase 3, catalytic subunit, alpha isozyme (PPP3CA), transmembrane 9 superfamily protein member 4 (TM9SF4), and short chain dehydrogenase/reductase family 16C, member 5 (SDR16C5). Similarly, the proteins with the largest numbers of neoepitopes are of interest. These include TP53, PTEN, as well as many olfactory receptors and follicular dendritic cell secreted protein (FDCSP). Of the Cancer Gene Consensus proteins (617, data accessed on June 28, 2017), 93.68% are among the proteins that yield many neoepitopes. Among these are polymerase (DNA directed), epsilon, catalytic subunit (POLE); PTEN; Fanconi anemia, complementation group A (FANCA); and CCR4-NOT transcription complex, subunit 3 (CNOT3) and others.

### Annotation of neoepitope-containing proteins

The variant dataset used in the study has been investigated previously. We have used PON-P2 variant tolerance/pathogenicity method to predict cancer-related harmful variants^22^. The performance of the method was verified on experimentally studied cancer variants. 14% of the variants were predicted to be harmful. We compared the three predicted categories of PON-P2, that is benign, harmful and variants of unknown significance, and found that the antigenicity and harmfulness were not correlated (data not shown). The harmful variants were not likely more antigenic than benign substitutions. This was expected as harmful variants affect crucial sites in proteins while neoantigens are cleaved peptides containing non-self characteristics.

Next, we characterized what kinds of proteins were enriched in neoepitopes. For this purpose, we collected the Gene Ontology^23^ annotations of all the human proteins and used them as the background. We sorted proteins containing neoepitopes and normalized them by the protein length (Table S2). We determined the enriched molecular function, biological process, and cellular component GO terms for the neoepitope-inducing proteins (Tables 3–5). The metabolic process (0008152) and cellular process (0009987) were the two main and related categories of biological process terms. The most specific terms at this level were related to nucleic acid metabolism (0090304) including RNA metabolic process (0016070). Other prominent categories were cellular protein metabolic process (0044260), gene expression (0010467) and macromolecule biosynthetic process (0009059).

The largest category of molecular function terms was binding (0005488), which was further divided into nucleic acid binding (0003676), protein dimerization (0046983), receptor binding (0005102), and protein complex binding (0032403), with more detailed daughter terms. The three other categories were nucleic acid binding (0001071), catalytic activity (0003824), and transporter activity (0005215). Transcription factor activity, RNA polymerase II distal enhancer sequence-specific binding (0003705) and oxidoreductase activity (0016712) were the most specific terms.

The enriched cellular component terms related to macromolecular complex (0032991), organelle (0043226), cell (0005623), and supramolecular complex (0099080). These were nested so that the most specific terms included only nucleosome (000786), nucleus (0005634), mitochondrion (0005739), and keratin filament (0045095).

To investigate the effect of AASs in neoepitopes or peptide characteristics, we compared the amino acid residue distributions for 8- to 11-mers. For this analysis, we concentrated on the positions of the AASs within the neoepitopes. The results in Fig. S6 show that the amino acid proportions for the variant substitutions and corresponding wild-type residues were clearly different. The results were visualized with MultiDisp that draws the characters based on the frequency in the data, i.e., the higher the occurrence, the taller the letter. Note that the figure does not show sequence context. Instead, it shows amino acid frequency data for each position within the n-mers.

Certain trends were evident and consistent throughout the different peptide lengths. The ratios of amino acid residues were similar with minor differences at the last positions for 10- and 11-mers. In the variant dataset enrichment of residues F, I, L, V, and Y was evident, especially in the last position of 8-, 9- and 10-mers (Fig. S6). The ratios of D, E, R, S, and T were reduced in neoepitopes compared to the distribution in the wild-type peptides. These trends were conserved at all positions but were very distinct at the last position.

The depleted residues are hydrophilic. These results are in line with a previous study^24^. The importance of hydropathy characteristics is related to the binding preference for amino acids within the HLA binding sites. Although, only some positions in HLAs are considered to be essential for recognition and response, our results showed that there were certain preferences at all sites. Overall, the positions in all n-mers were more hydrophobic in neoepitopes than in wild-type peptides (Fig. S7). The largest differences were in the last position for 8-, 9- and 10-mers.

Hydropathy is a fundamental property of molecules. However, it is difficult to estimate. There are many scales for residue hydropathy. The AAindex database contains over 100 such propensity scales^25^. We have shown that there are differences in the prediction performance of the scales^26^. To be able to compare our results to those published earlier, we used the Kyte-Doolittle hydropathy scale^27^.

## Discussion

Our analysis indicated that neoepitopes are very frequent in all cancers. We used the NetMHC program for the predictions. This tool has been widely used and has behaved favorably in method assessments^28^. However, it is obvious that the tool overpredicts. In addition to variant peptides, large numbers of wild-type peptides were predicted to be antigenic, which cannot be possible. The production of self-recognizing antibodies is tightly controlled to avoid autoimmune reactions and diseases.

To concentrate on the most likely neoepitopes, we defined them as having high binding affinity for the variants and not binding or having low binding affinity for the wild-type peptide. After this filtering, we had 0.24% of the original peptides left. This still accounts for 11,316,078 neoepitopes. These are very evenly distributed to peptide positions and HLAs. 9-mers are clearly the most prominent among neoepitopes accounting for altogether 72.26% of neoepitopes (Fig. 3).

The enrichment analysis of GO terms indicated binding and metabolic processes to be important. Molecular functions of proteins containing neoepitopes included terms for binding, catalytic and transporter activities. Additionally, numerous cellular compartments were enriched.

After filtering, we still retained neoepitopes for 89.15% of human proteins. It is evident that all the filtered neoepitopes cannot be biologically functional. The NetMHC tool has its limitations which emerge from the complexity of the T cell response and from the lack of the full understanding of the many details of this process. Numerous factors contribute to T cell activation. Peptide binding to MHC molecules is just one of them. These processes have been discussed, e.g. in^29,30^.

Peptide binding to an HLA molecule is a requirement for raising adaptive immunity. This is dependent on the sequence but also on the processing of the precursor protein in the antigen presenting cells by the proteasome and other proteases. The peptide is transported from the cytosol to the endoplasmic reticulum by the transporter associated with antigen processing (TAP) complex of TAP1 and TAP2 proteins. The transport is based on ATPase activity. To prevent wasting ATP, the transporter selects for high-affinity peptides.

Although a peptide may have high affinity, it may not be an efficient epitope due to, e.g., low abundance and low stability. Further, the peptide-MHC complex has to be recognized by T cells as immunogenic. Recently, methods to define the specificity of the T cell receptors have been presented^31,32^. Self-recognition systems prevent the production of antigens recognizing epitopes too similar to natural human proteins. It has been estimated that one-third of the peptides are too similar to self^29^. Thus, only a fraction of the neoepitopes can elicit an immune response.

Experimental data is available for very small numbers of tested peptides. Studies on two HLAs in binding vaccinia virus peptides indicated that about 2.5% of all 9- and 10-mer peptides bind with affinity ≤100 nM^33^. This is in line with our results that indicated weak and high binders to account for 2.8% of all the peptides. Our threshold for high binding peptides was 50 nM and 500 nM for low binders.

When the high binding peptides for vaccinia virus were tested experimentally, only 56% were immunogenic^33^. Peptide immunization studies revealed that 15% of the peptides elicited T cell response capable of recognizing vaccinia virus infected cells. However, only 15 (11%) were immunodominant and recognized during virus infections. In conclusion, only 0.89% of the high binders were true epitopes. When this ratio is applied to our data, there would be 101,097 neoepitopes, which is still a substantial number and indicates a high potential for neoantigen-based treatment and diagnosis.

Although neoepitopes and their usage in clinical applications have been discussed^34^, there are also notes that immunogenic neoepitopes are exceptions rather than the norm^35^. We can further correlate our findings to the knowledge on T cell response. Analysis of vaccinia virus WR strain indicated that only 49 epitopes accounted for 94.8% of the CD8+ cell response^36^. They predicted in total 175,458 of 8-, 9- and 10-mer peptides. For the experimental studies, 2256 peptides were chosen. If we use the same ratio of peptides (49/175,458) to estimate the number of effective peptides in our datasets, we will obtain about 1.3 million peptides.

Humans have six HLA genes. Currently, the major database for HLA alleles^37^ contains 12,351 HLA class I alleles for different ethnic groups. The most common alleles are very frequent, and therefore our results apply to a large part of populations.

It is likely that neoantigens that are recognized by several HLAs and with different peptide lengths, denoted here as super-epitopes, also raise the T cell response at least in some cases. Thus, the variants that are predicted to be neoepitopes numerous times are the top candidates for neoantigen therapy and other applications. Our top candidates are 1282 variants which were predicted to be antigenic at least 100 times.

Recent phase I clinical studies with neoantigen vaccines^38,39^ were very promising and indicated that peptides representing 10 to 20 neoantigens presented good safety and efficacy when tested on stage III and IV melanoma patients. The neoantigens selection is a crucial issue both for efficacy as well as for preventing immunotoxicity and autoimmunity^40^. Endeavors like the Human Immunome Peptidome Project Consortium^41^ and Tumor Neoantigen Selection Alliance will provide essential contributions.

Immunotherapies are promising but can cause increased progression in some cases such as PD-1 inhibitors in melanoma and lymphoma. Therefore, special care has to be taken for the selection of therapy, including neoantigen vaccines^42,43^.

Processing, recognition, transport and binding affinity are important for immunogenicity. Apart from peptide selection, the delivery of the peptides and adjuvant selection are crucial for vaccination. It is likely that immunotherapy should be combined with other forms of therapy, including surgery, radiation and chemotherapy.

As more experimental data become available the quality of prediction methods that take advantage of these data shall improve. Better prediction tools and improved experimental validation assays will lead to improvements in clinical and other applications of neoantigens including personalized medicine and diagnosis.

## Methods

### Variations and HLA binding affinity prediction

The AASs in 30 cancer types were obtained from^4^. The sequences and annotations of the longest transcripts for each protein were retrieved from the Ensembl biomart release 69 repositories.

For each AAS in the dataset, we constructed a 21-mer peptide. In the middle of the peptide, at position 11, was the substitution position. This residue was flanked by ten amino acids on both sides. Two such sequences were constructed per AAS, one for the wild-type and the other for the variant sequence. Both sequences were in fasta format. These peptides allowed predictions to be made for the variant at all possible positions in peptides of lengths 8, 9, 10, and 11. The peptide sequences were concatenated and used as input in the NetMHC 4.0 epitope-HLA prediction algorithm^10^ that was run locally. The prediction results for each HLA were stored in tab-separated files. Peptides predicted to be neither weak nor strong binders were filtered out using bash commands and R scripts.

To identify neoepitopes, we compared the predicted affinities for the wild-type and corresponding variant-containing peptides. Peptides were defined as neoepitopes for a certain HLA molecule when the variant was predicted to bind strongly whereas its wild-type form was predicted to bind weakly or not at all. Variant peptides with affinity IC_50_ < 50 nM were classified as high binders, those with 50 < IC_50_ < 500 nM as weak binders, and peptides with IC_50_ > 500 nM as non-binders.

### Data analysis

Data manipulations and analyses were performed in R, a statistical analysis and programming software environment^44^. The amino acid and hydropathy data were visualized with MultiDisp (http://structure.bmc.lu.se/MultiDisp), a web program for analyzing multiple sequence alignments. Kyte and Doolittle^27^ propensities were used to calculate hydropathy values with a sliding window method.

The enrichment of GO terms for proteins from which the neoepitopes were obtained was calculated with GOrilla^45^. The web service uses the minimum hypergeometric method that computes the hypergeometric statistic of the top *k* ranked elements of a vector^46^. The top *k* elements were selected to optimize enrichment^45^. REViGO^47^ was used to remove redundant terms and to summarize the terms. It defines semantic similarity for pairs of GO terms based on their shared parent terms and visualizes the results for GO term enrichment from GOrilla.

## Electronic supplementary material

Supplementary Figures

Table S1. The list of variants with the largest number of neoepitopes

Table S2. The proteins that yield the largest number of neoepitopes

Table S4. Results of Gene Ontology term enrichment for molecular function analysis of proteins that yield larger numbers of neoantigens

Table S3. Results of Gene Ontology term enrichment for biological process analysis of proteins that yield larger numbers of neoantigens.

Table S5. Results of Gene Ontology term enrichment for cellular compartment analysis of proteins that yield larger numbers of neoantigens

## Data Availability

The datasets generated and/or analyzed during the current study are not publicly available due their sizes but are available from the corresponding author on reasonable request. The data generated and analyzed in the current study was retrieved from the Ensembl biomart release 69 repositories and from^4^.
