# Supplementary material for: Pan-cancer analysis of neoepitopes
Source: Sci Rep. 2018 Aug 24;8:12735. doi: 10.1038/s41598-018-30724-y (PMC6109115; doi:10.1038/s41598-018-30724-y)
Supplement: Supplementary file 1 — Supplementary Figures [file 41598_2018_30724_MOESM1_ESM.pdf]

Pan-cancer analysis of neoepitopes

Gabriel N. Teku and Mauno Vihinen

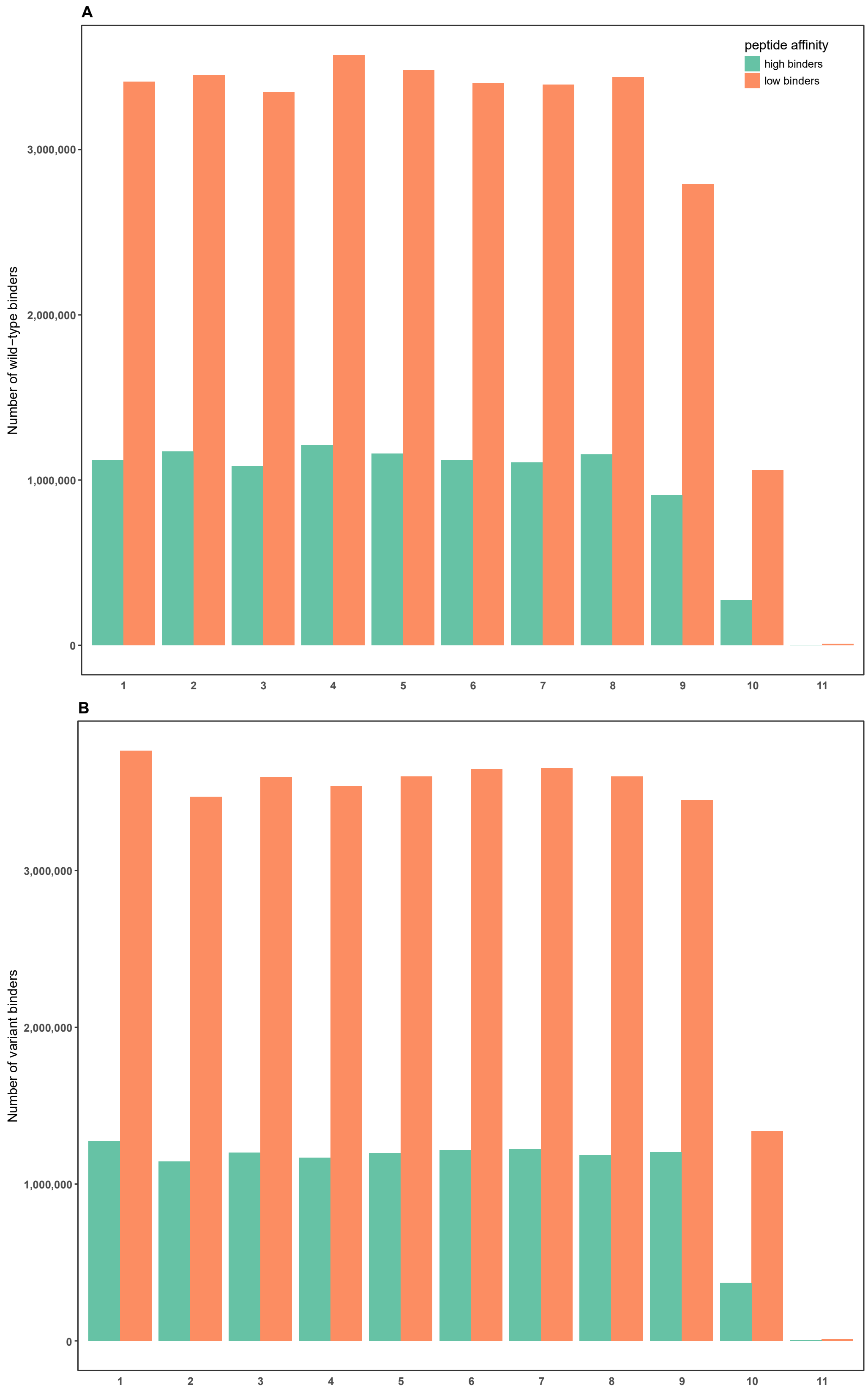

A
peptide affinity
high binders
low binders
Number of wild−type binders
3,000,000
2,000,000
1,000,000
0
1
2
3
4
5
6
7
8
9
10
11
B
Number of variant binders
3,000,000
2,000,000
1,000,000
0
1
2
3
4
5
6
7
8
9
10
11

Supplementary Figure 1. The distribution of AASs at positions 1-11 in the predicted wild-type and variant HLA binders. The distribution of AASs to positions 1-11 on both the wild-type, A, and variant peptides, B, and in both the high binders (green) and weak binders (red) are quite similar.

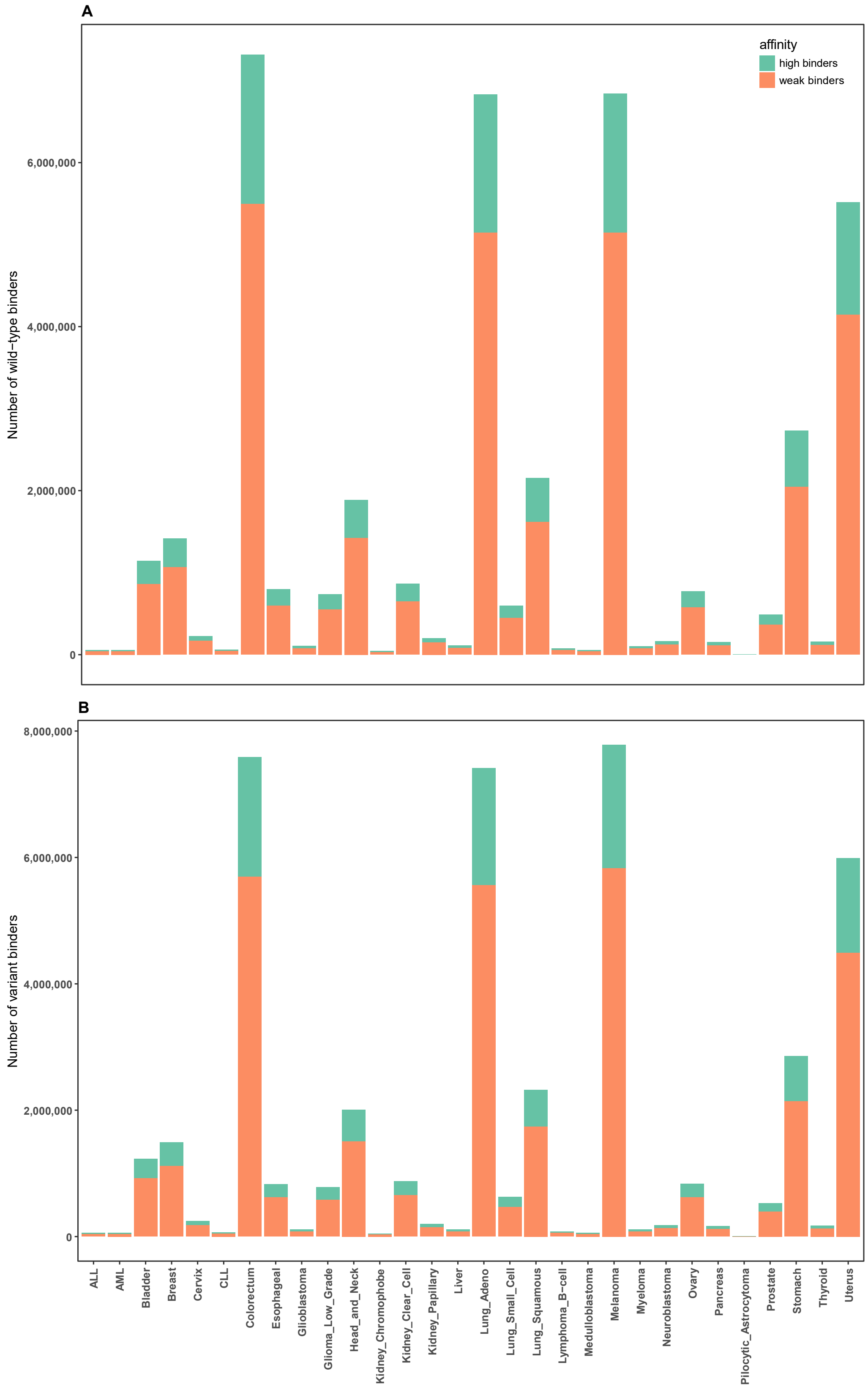
A
B
affinity
high binders
weak binders
Number of wild-type binders
Number of variant binders
0
2,000,000
4,000,000
6,000,000
8,000,000
ALL
AML
Bladder
Breast
Cervix
CLL
Colorectum
Esophageal
Glioblastoma
Glioma_Low_Grade
Head_and_Neck
Kidney_Chromophobe
Kidney_Clear_Cell
Kidney_Papillary
Liver
Lung_Adeno
Lung_Small_Cell
Lung_Squamous
Lymphoma_B-cell
Medulloblastoma
Melanoma
Myeloma
Neuroblastoma
Ovary
Pancreas
Pilocytic_Astrocytoma
Prostate
Stomach
Thyroid
Uterus

Supplementary Figure 2. The distribution of wild-type and variant high and low binders across cancer types.

The proportions of wild-type (A) and variant binders (B) across the cancer types. There are no differences in the distributions of binders across both the wild-type and variant peptides, per cancer type.

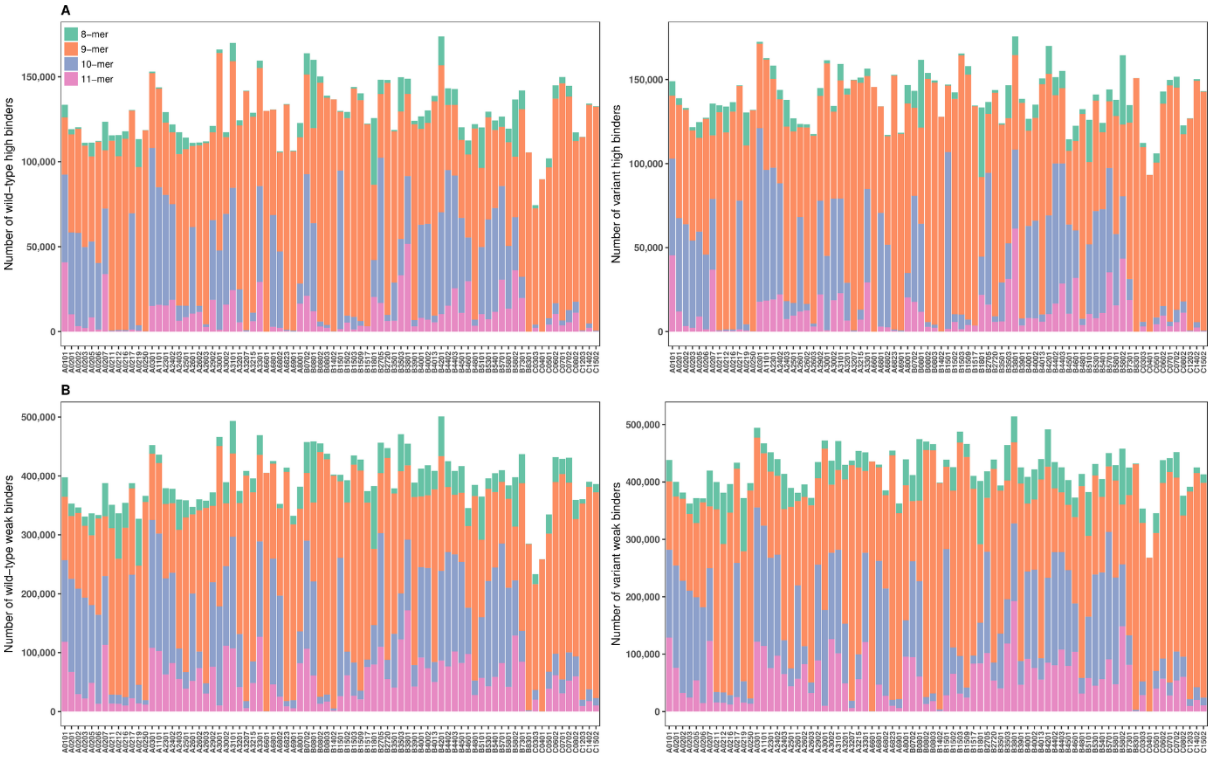

Supplementary Figure 3. The distribution of wild-type and variant high and low binders across HLAs.

(A) The distribution of high binders in the wild-type (top left panel) and variant datasets (top right panel). (B) The distribution of weak binders in the wild-type (bottom left panel) and variant datasets (bottom right panel).

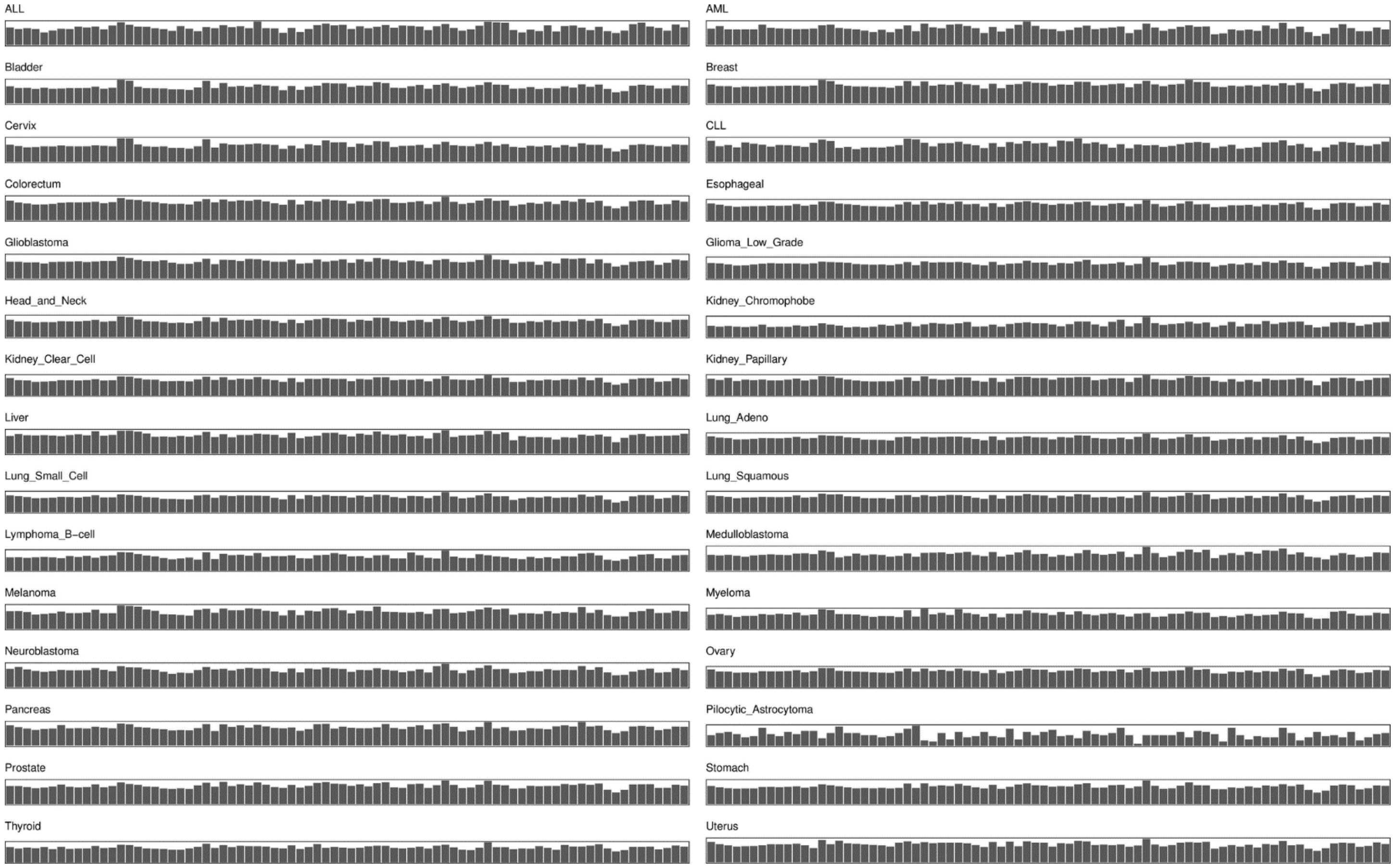

Supplementary Figure 4. Cancer-specific neoepitope distributions across HLAs.

The distributions across the cancer types are overall very similar. This indicates differences in HLAs irrespective of the total numbers of variants, which vary very widely.

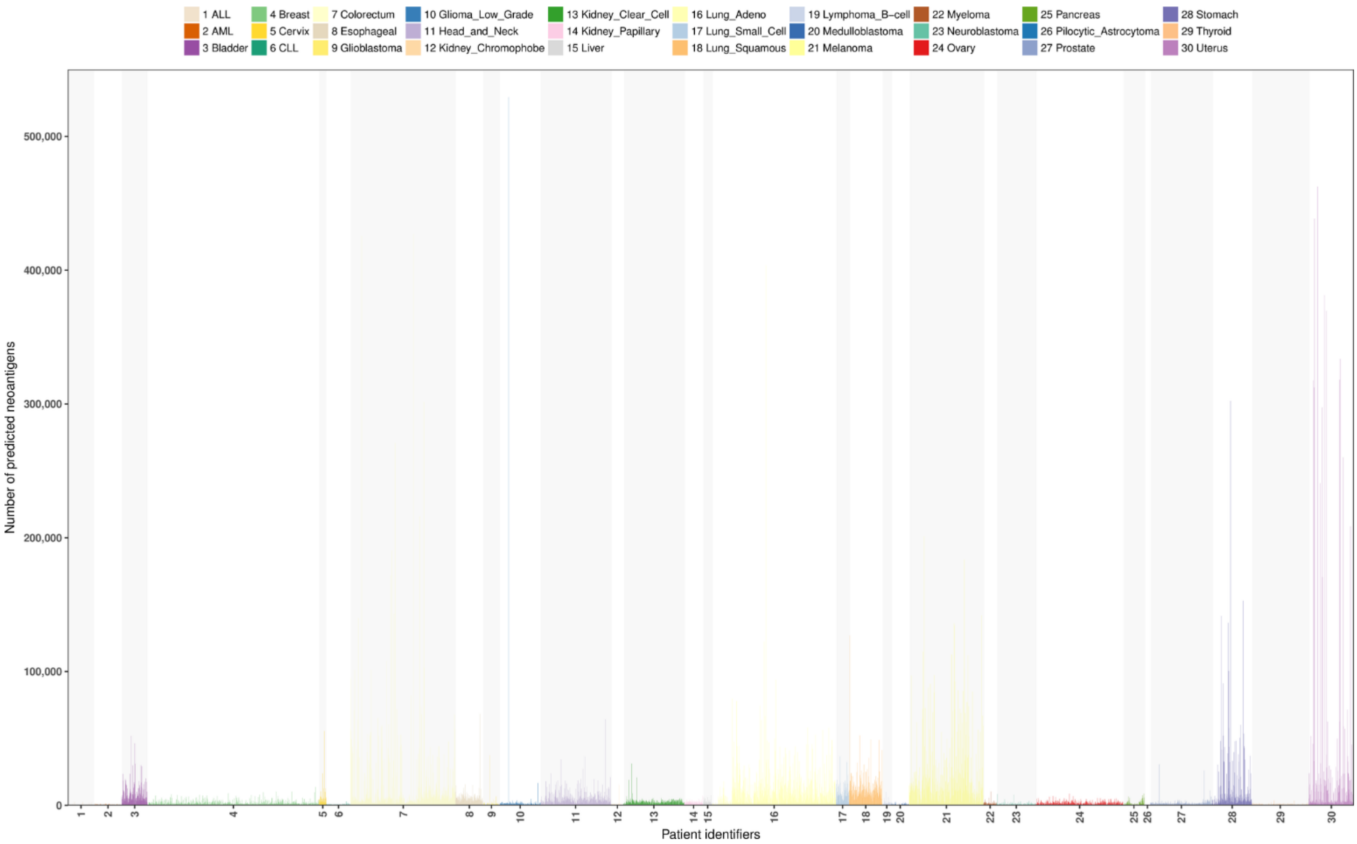

Supplementary Figure 5. Distribution of neoepitopes across cancer patients.

Each color and number are a cancer types. The height of the lines represents the abundance of a cancer type in the patient. Each patient is represented on the x-axis and group/colored by cancer type. The differences in neoepitope numbers among patients in the cancer types are high. The minimum and maximum numbers of neoepitopes per patient are 4 and 529,280, respectively.

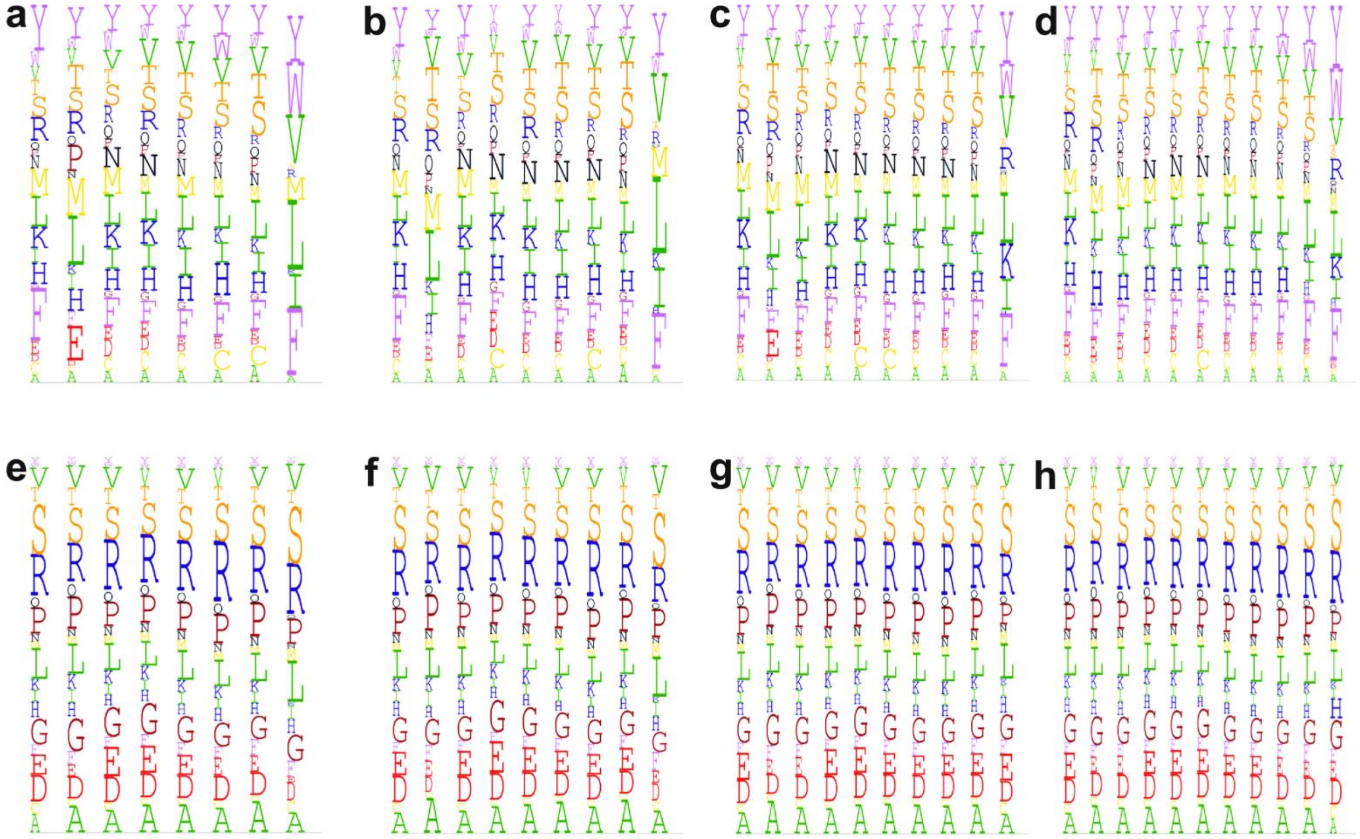

Supplementary Figure 6. The amino acid substitution frequency at the amino acid substitution position.

The upper and the lower row contain the variant and wild-type data, respectively. Panels a, b, c, and d consist of neoantigen 8-, 9-, 10 and 11-mer data respectively (top panels), while panels e, f, g, and h, consist of wild-type 8-, 9-, 10 and 11-mer data, respectively. . The results are visualized with MultiDisp that draws the characters based on the frequency in the data, i.e., the higher the occurrence, the taller the letter. The figure shows amino acid frequency data for each position within the n-mers.

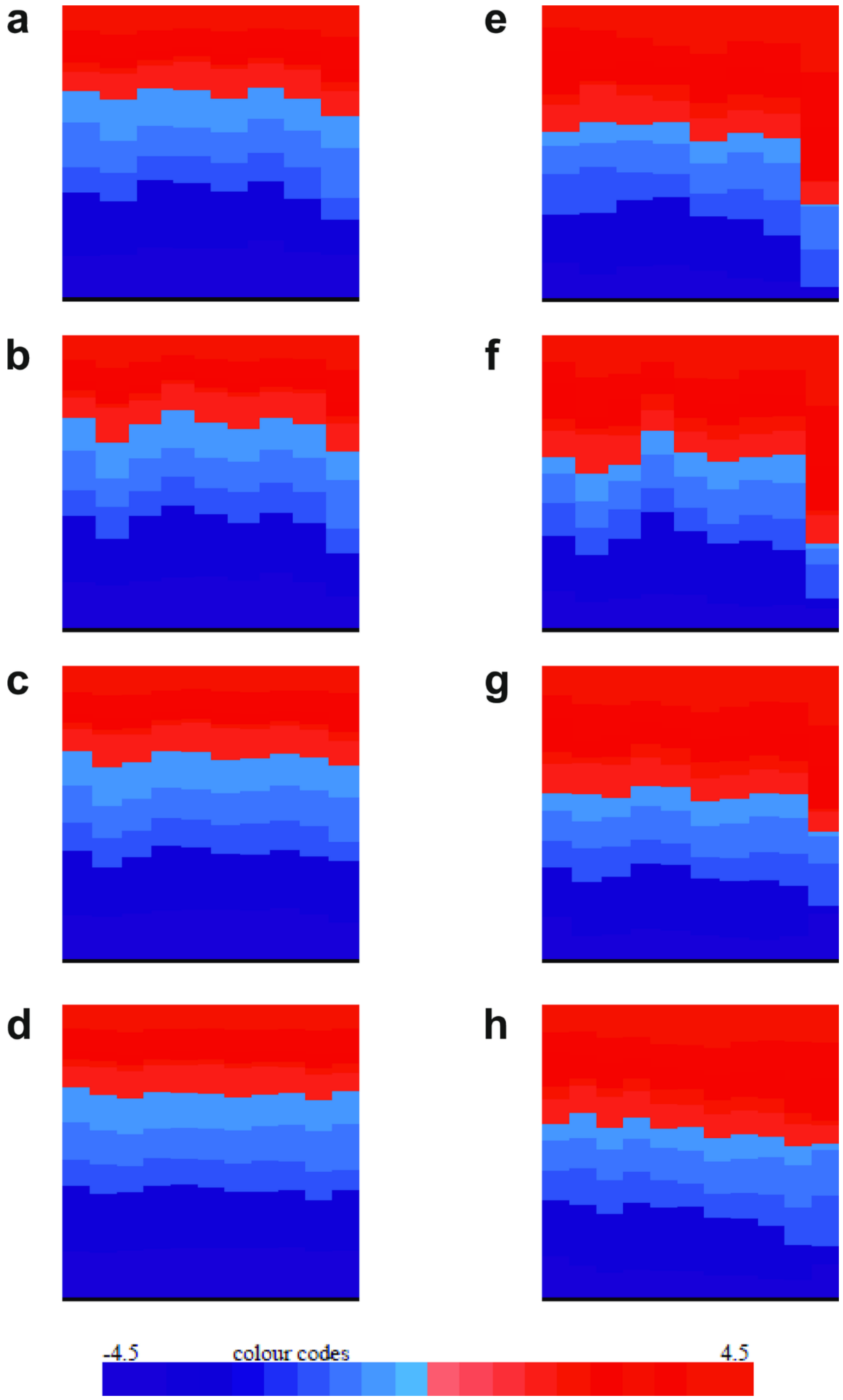

Supplementary Figure 7. Hydropathy of amino acid residues at the substitution position on the n-mers.

The panels on the left are hydropathy predictions for the neoepitopes, while those on the right are for wild-type peptides. From top to bottom, panels a, b, c, and d, represent 8-, 9-, 10- and 11-mer neoantigen data, while panels e, f, g, and h, are for wild-type.
